# Is Shoulder Pain and Disability Index a Prognostic Factor for Neuropathic Shoulder Pain?

**DOI:** 10.7759/cureus.19173

**Published:** 2021-10-31

**Authors:** Sotiria D Vrouva, Varvara K Sopidou, Konstantinos P Chanopoulos, Daphne F Bakalidou, Vasileios C Papatsimpas, Nikolaos Sorras, Miltiades C Ziogas, George A Koumantakis

**Affiliations:** 1 Physical Therapy/Pain Management, 401 General Army Hospital of Athens, Athens, GRC; 2 Laboratory of Neuromuscular and Cardiovascular Study of Motion (LANECASM) Physiotherapy Department, Faculty of Health and Care Sciences, University of West Attica, Athens, GRC; 3 Department of Biomedical Sciences, Faculty of Health and Caring Professions University of West Attica, Athens, GRC; 4 Computational of Mathematics and Decision Making, 401 Army General Hospital of Athens, Athens, GRC; 5 Pain Management, 401 Army General Hospital of Athens, Athens, GRC; 6 1st Department of Orthopedics, 401 Army General Hospital of Athens, Athens, GRC; 7 Physiotherapy Department, School of Health and Care Sciences, University of West Attica, Athens, GRC

**Keywords:** shoulder, spadi, neuropathic pain, disability, rotator cuff

## Abstract

Introduction

So far, investigations in patients with rotator cuff diseases have used pain measurement tools such as visual analog scale (VAS) for nociceptive pain as well as neuropathic pain (NeuP) specialized ones like Douleur Neuropathique 4 Question (DN4) and Pain Detect. The study’s goal was to look at the existence of NeuP in patients with chronic shoulder pain, as well as variables that may be predictive of its progression.

Methods

There were 112 outpatients in all. Current and previous pain intensity levels were documented with the numerical rating scale (NRS), the Shoulder Pain and Disability Index (SPADI) was used to assess pain and disability levels, and the S-LANSS (self-completed Leeds Assessment of Neuropathic Symptoms and Signs Pain Scale) was used to diagnose NeuP. The Pearson Chi-Square test was employed to check for any relationships between variables. The Mann-Whitney U test was also employed to check for between-group differences (with or without NeuP). To investigate factors that may be utilized as a prognostic for NeuP, logistic regression was performed, with those components (from the univariate analysis) that were statistically significant being included.

Results

According to the S-LANSS questionnaire for NeuP diagnosis, 21 patients had NeuP. According to S-LANSS, chi-square test findings revealed that NeuP is independent of sex, smoking, size, and location or rotator cuff tear. Univariate analysis with Mann-Whitney U test revealed statistically significant differences in SPADI and NRS scores between the two patient groups (p < 0.001). Α multivariate analysis using S-LANSS as the binary dependent variable and NRS currently, NRS average last month and SPADI total score as independent variables (with statistical significance) revealed that total SPADI score may be considered as an independent prognostic factor for NeuP (odds ratio = 1.189, p < 0.001).

Limitations

Due to the limited number of patients who participated in the study, the findings were deemed insufficient in terms of statistical power. In particular, the power analysis of the study (type I error probability being [a] = .05) was less than 80% (for the total SPADI score), hence relatively small. As a result, there is a limited probability of a type I error.

Conclusions

Using S-LANSS, we discovered that 18.8% of patients with rotator cuff tears had NeuP. The SPADI scores (pain and disability) in the NeuP group were substantially greater than in the nociceptive pain group. As previous studies have suggested utilizing certain levels of the VAS for pain assessment and specialized questionnaires for NeuP evaluation, we recommend that SPADI be included as a tool for emphasizing the neuropathic features of shoulder pain.

## Introduction

Globally, there has been a trend in study toward identifying variables that might emphasize the various aspects of neuropathic pain (NeuP) in musculoskeletal disorders because this will affect prognosis and treatment [1]. A systematic review on shoulder pain, in particular, found that studies are demonstrating a central sensitization component in shoulder pathologies [1]. Moreover, NeuP characteristics have been discovered in a range of shoulder pathologies, including rotator cuff diseases and glenoid labrum ruptures [2-6]. As a result, more research into the features of NeuP in this group is needed.

This study aimed to investigate the existence of NeuP in patients with a rotator cuff tear, as well as factors that may be prognostic of NeuP development. So far, investigations in patients with rotator cuff diseases have employed pain measurement tools like visual analog scale (VAS), as well as more NeuP specific ones like Douleur Neuropathique 4 Question (DN4) and Pain Detect [6,7]. To the best of our knowledge, no studies have investigated whether a questionnaire such as Shoulder Pain and Disability Index (SPADI) might be used as a prognostic factor for the development of NeuP in chronic shoulder pain.

## Materials and methods

The present study involved patients who had consulted with an orthopedic surgeon for their shoulder pain and were scheduled for surgery between March 2016 and March 2019, with a referral for pre-operative physiotherapy (Approval of Ethical Committee of 401 General Army Hospital of Athens N EBΔ 624/11-2-15). The patients provided their written informed consent for the collection of their data and the research complied with all principles of the Helsinki Declaration. 

Outpatients with >3 months of chronic shoulder pain of a non-traumatic origin were included in the study (inclusion criteria). Conversely, patients with previous shoulder surgery, rheumatic diseases, cancer, mental disorders, neurological pathologies, cardiovascular diseases, and the use of painkillers were excluded from the study (exclusion criteria).

**Figure 1:**
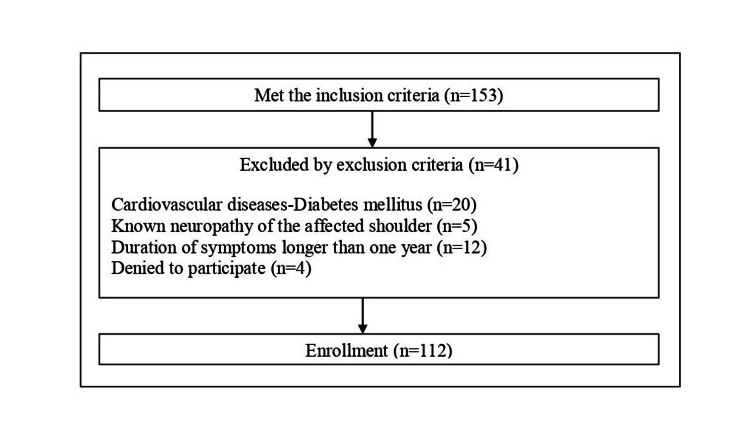
Flow chart with the number of patients enrolled.

Data from 153 patients were gathered, 37 of whom did not meet the admission criteria, while four refused to participate (Figure 1). Finally, the study was performed with 112 patients; the patients were asked to indicate their age, gender, smoking habits, as well as to complete the numerical rating scale (NRS) scale (at present and over the past month on an average), the SPADI questionnaire, and the S-LANSS scale for NeuP. Data were consecutively collected from patients, with no attempt at randomization.

The S-LANSS

S-LANSS (self-completed Leeds Assessment of Neuropathic Symptoms and Signs Pain Scale) is a reliable and valid tool, composed of five general questions related to the characteristics of pain and two related to self-evaluation of pain by the patients [8]. The score ranged from 0 to 24, and values >12 indicated NeuP [8,9]. The S-LANSS questionnaire’s sensitivity was 78% for the critical value of 12 [8]. The scale had also been validated in the Greek language while retaining the original measurement properties [9].

The SPADI

SPADI (Shoulder Pain and Disability Index) is a self-administered questionnaire suitable to investigate shoulder pain and related dysfunction [10]. It comprised 13 questions, with the first 5 measuring pain and the remaining 8 assessing the patients’ disability [10]. Internal consistency for SPADI in patients with a rotator cuff was found to be quite high (Cronbach’s alpha was 0.932) among the Greek population [11]. Therefore, it can be used as a valid and reliable tool for recording pain and incapacity caused by shoulder pain in patients, especially when combined with the NRS [10].

NRS

The NRS (numerical rating scale) is one of the most commonly used pain scales in medicine [12,13]. NRS consists of a numeric scale similar to the VAS and less affected by non-pain intensity factors [12,13]. The study patients were asked to evaluate their pain both during the initial visit and on an average during the last month, in a range of 0 to 10, with zero being an example of someone with no pain and 10 indicating the worst possible pain. NRS evaluates pain experienced as “an average pain intensity” [14].

MRI

Magnetic resonance imaging (MRI) examination was performed and the scans were collected for all patients showing symptoms of shoulder pain. The scans were then inspected to assess the size and the position of the rotator cuff tear, suggesting high sensitivity and specificity (91.1%) [15].

Statistical analysis

To discover the possible dependencies in patients with a rotator cuff tear, between NeuP and qualitative variables (i.e., gender, smoking, tear size, and location), we used the Pearson Chi-Square test. In addition, the Mann-Whitney U test was performed to test the differences between the two groups (with or without NeuP). In order to assess whether the SPADI score could be used as a prognostic factor for NeuP, logistic regression was performed using the statistically significant factors that emerged from the univariate analysis. Statistical analyses were performed by using the IBM SPSS Statistics v.22 for Windows (SPSS Inc. Chicago, IL, USA), with the statistical significance for all tests set to 0.05. In addition, a power analysis was performed with the GPower 3.1.9.4, to calculate the post-hoc power required for any statistical relation to be established as significant.

## Results

Table 1 shows the characteristics of the patients (demographic data, clinical features) who were recruited in the trial (n = 112) based on inclusion/exclusion criteria, including age, gender, smoking, SPADI scores, and NRS pain scores.

**Table 1:** Characteristics of patients enrolled in the study (n = 112). ^a^Data are presented in the form of mean ± SD (range). ^b^Data are presented in the form of no. of patients. SPADI: Shoulder Pain and Disability Index.

Age^a ^(years)	53.35 ± 10.829
Gender (male)^b^	47
Gender (female)^b^	65
Smoking ^b^	70
No smoking ^b^	42
SPADI pain score^a^	62.64 ± 16.617
SPADI disability score^a^	43.58 ± 18.565
SPADI total score^a^	50.83 ± 16.793
Numerical rating scale now^a^	5.46 ± 1.963
Numerical rating scale average last month^a^	5.84 ± 1.984
Size of rotator cuff tear (small)^b ^(mm)	35
Size of rotator cuff tear (medium)^b ^(mm)	74
Size of rotator cuff tear (large)^b ^(mm)	3
Rotator cuff tear partial sided (articular)^b^	36
Rotator cuff tear partial sided (bursal)^b^	58
Rotator cuff tear partial sided (intratendinous)^b^	18
Tear location (supraspinatus)^b^	98
Tear location (infraspinatus)^b^	10
Tear location (subscapularis)^b^	4

The findings of the MRI assessment revealed that 31.3% (n = 35) had a first-grade tear (<3mm deep), 66.1% (n = 74) a second grade (3-6mm deep), and only 2.7% (n = 3) were categorized as having a third-grade tear. As far as the side of tear was concerned, 32.1% (n = 36) were articular, a 51.8% (n = 58) were bursal, and 16.1% (n = 18) were intratendinous. In terms of tear location, the majority of patients, 87.5% (n = 98), had the tear in the supraspinatus, followed by 8.9% (n = 10) in the infraspinatus, and lastly 3.6% (n = 4) in the subscapularis.

Twenty-one patients (18.8%) were assigned to the NeuP group according to the S-LANSS score (≥12), while 91 patients (81.3%) were assigned to the nociceptive pain group. According to SLANSS questionnaire data, NeuP is independent of sex, smoking, size, and location or rotator cuff tear (Table 2).

**Table 2:** Categorical factors associated with neuropathic pain (n=112). Data are presented in the form of % value of patients. NeuP is likely with S-LANSS score ≥ 12. NeuP: neuropathic pain; S-LANSS: self-completed Leeds Assessment of Neuropathic Symptoms and Signs Pain Scale.

Factor	Neuropathic pain (NeuP)	Nociceptive pain
Gender (male)	42.9	41.8
Gender (female)	57.1	58.2
Smoking	33.3	38.5
No smoking	66.7	61.5
Size of rotator cuff tear (small) (mm)	28.6	31.9
Size of rotator cuff tear (medium) (mm)	71.4	64.8
Size of rotator cuff tear (large) (mm)	0	3.3
Rotator cuff tear partial sided (articular)	38.1	30.8
Rotator cuff tear partial sided (bursal)	47.6	52.7
Rotator cuff tear partial sided (intratendinous)	14.3	16.5
Tear location (supraspinatus)	90.5	86.8
Tear location (infraspinatus)	9.5	8.8
Tear location (subscapularis)	0	4.4

On the other half of univariate analysis, Mann-Whitney U test results revealed statistically significant differences in SPADI and NRS scores between the two groups of patients. The NeuP group’s mean NRS scores (now and average last month) were significantly higher (p-values < 0.001). Similarly, the NeuP group’s mean SPADI scores (pain, disability, and total) were significantly greater than the nociceptive pain group (Table 3).

**Table 3:** Differences between the neuropathic pain (NeuP) and nociceptive pain groups (n = 112). Data are presented in the form of mean ± SD (range). NeuP is likely with an S-LANSS score ≥12. p < 0.05 indicates statistically significant difference. S-LANSS: self-completed Leeds Assessment of Neuropathic Symptoms and Signs Pain Scale.

Variable	Neuropathic pain (NeuP) (n = 21)	Nociceptive pain (n = 91)	p-value
Age (years)	52.38 ± 10.443	53.57 ± 10.961	0.451
Numerical rating scale now	6.90 ± 1.640	5.13 ± 1.887	0.000
Numerical rating scale average last month	7.29 ± 2.101	5.51 ± 1.810	0.000
SPADI pain score	79.05 ± 10.519	58.86 ± 15.449	0.000
SPADI disability score	65.33 ± 11.342	38.56 ± 16.145	0.000
SPADI total score	70.67 ± 10.209	46.25 ± 14.548	0.000

The results of multivariate analysis, multiple logistic regression, with S-LANSS as the binary dependent variable and NRS ''now'', NRS average last month, and SPADI total score as independent variables with statistical significance, revealed that the total SPADI score can be regarded as an independent prognostic factor for NeuP (p-value < 0.001) in contrast to the other variables (Table 4).

**Table 4:** Multiple logistic regression results with S-LANSS as the binary dependent variable. Statistically significant difference (p < 0.01). NRS: numerical rating scale; SPADI: Shoulder Pain and Disability Index; S-LANSS: self-completed Leeds Assessment of Neuropathic Symptoms and Signs Pain Scale.

Independent variable	Exp(B)	95% confidence interval	p-value
NRS now	1.111	0.675-1.830	0.678
NRS average last month	1.050	0.671-1.645	0.830
SPADI total score	1.189	1.089-1.298	0.000

The model correctly classified 88.4% of cases that confirm the effectiveness of the predicted classification against the actual classification. Furthermore, the model fits the data well (Hosmer-Lemeshow test, p = 0.091), and, with an odds ratio for total SPADI score (Exp(B) = 1.189) greater than 1, it implies that the possibility under research is more likely to occur in the first group of NeuP.

## Discussion

The S-LANSS was selected as a tool since it can detect NeuP and has high sensitivity and specificity [9], without the requirement for a clinical examination [8]. According to Bennet et al. [8], it may also be used to evaluate patients who have so-called mixed pain (nociceptive and neuropathic), such as patients with rotator cuff tears. Moreover, the validation sample includes patients with chronic musculoskeletal disorders [9].

It is frequently difficult to pinpoint the specific location of common shoulder discomfort since it tends to spread. According to Bayam et al [16], discomfort on the anterior and dorsal surfaces of the arm is associated with rotator cuff diseases. This corresponds to the pain distribution shown by 86 of the patients on the homunculus diagram contained in S-LANSS, which includes the upper trapezius area as well [16]. Although NeuP differs from nociceptive pain [17], it appears that they both employ the same nociceptive baseline mechanisms [18]. Due to peripheral and central sensitization, low-intensity harmful stimuli, as well as stimuli such as touch [19] and pressure [2], cause pain in NeuP [20,21].

Chronic shoulder diseases, as described by Bayam et al [16], produce a “burning sensation,” “pins and needles discomfort,” and stabbing-shooting pain. However, despite being mentioned, there are no reports of NeuP characteristics. Patients in our research reported having these traits, despite having an S-LANSS score of less than 12.

Additionally, 25.9 % (n = 29) described their pain as tingling, while only 2.7 % (n = 3) noticed a change in shoulder color, 26.8% (n = 30) were excessively sensitive to pain, 25.9% (n = 29) felt “electric shocks,” 25.0% (n = 28) a shooting pain, 3.6% (n = 4) a different friction sensation, and 17.0% (n = 19) a different contact sensation.

Patients with rotator cuff tears, in particular, exhibit neuropathic features [6-7,22]. Relevant reports for patients with NeuP in rotator cuff ruptures demonstrate percentages of 10.9 % [20] and 15.8% [6], respectively, but in our study, the proportion climbed to 18.8%.

Furthermore, the presence of NeuP corresponds with higher SPADI scores, despite the fact that in many studies, high VAS pain rates appear to be related to the presence of NeuP [23]. The main concern with NeuP is not tissue destruction, but rather the presence of pain and how it impacts functioning. This is more observable with SPADI, which may assess impairment in addition to pain, rather than with VAS, which just displays pain levels. Luque-Suarez et al. [24] demonstrated that greater scores of SPADI are associated with decreased range of motion in chronic unilateral shoulder pain. MRI, smoking, and sex data were also included in the univariate analysis; however, unlike prior studies [6,7], they did not appear to correlate with the group in which NeuP was detected.

The developer of the SPADI scale recommends it as a tool for measuring both pain and functional discomfort. Although it proposes five issues for pain and eight for disability, several studies have reached differing findings of whether questions favor the first or the second factor [25-27]. There have been studies that show that the characteristics of pain are the primary reason prohibiting various patients’ activities [28,29]. This might lead to the observation that, while our patient sample might have functional limitations, they may also be more sensitive to pain perception. Due to the above, the total SPADI score, rather than its subsets individually, proved statistically significant (SPADI pain and disability).

According to this study, one unit increase in SPADI total score increases the odds of having NeuP by 19% when compared to patients who scored low in SPADI. While Haampaa et al. [30] propose VAS for measuring pain intensity and special questionnaires for NeuP assessment, we might incorporate SPADI as a tool for highlighting the neuropathic features of shoulder pain based on our findings.

Given that the existence of NeuP may influence the outcome of the operation [3,6], it would be important to determine if the pain will persist after the anatomy of the region is restored.

Limitations

The statistical power of the findings was deemed inadequate due to the limited number of patients who participated in the research. The power analysis for the study (type I error probability being [a] = .05) was less than 80% (for the total SPADI score), suggesting that the sample size was small. As a result, the likelihood of committing a type I error is low. We propose performing more studies on a larger sample size to confirm the current findings. Aside from that, this study relied on self-reported assessments of pain and impairment levels; however, future studies should incorporate a clinical objective evaluation (such as a measure of motion range).

## Conclusions

We discovered that 18.8 % of patients may have NeuP by utilizing the S-LANSS as a specialized questionnaire for NeuP screening. Furthermore, multiple logistic regression revealed that the SPADI total score may be used safely as a prognostic factor for NeuP in patients with chronic pain due to rotator cuff tears. The SPADI scores (pain and disability) in the NeuP group were substantially greater than in the nociceptive pain group. As previous studies have suggested utilizing certain levels of the VAS for pain assessment and specialized questionnaires for NeuP evaluation, we recommend that SPADI be included as a tool for emphasizing the neuropathic features of shoulder pain.
